# Omitting Elective Irradiation of the Contralateral Retropharyngeal Nodes in Oropharyngeal Squamous Cell Carcinoma Treated with Intensity-modulated Radiotherapy

**DOI:** 10.7759/cureus.3825

**Published:** 2019-01-04

**Authors:** Moeko Nagatsuka, Ryan T Hughes, Rachel F Shenker, Bart A Frizzell, Kathryn M Greven

**Affiliations:** 1 Radiation Oncology, Wake Forest School of Medicine, Winston-Salem, USA

**Keywords:** oropharyngeal cancer, retropharyngeal lymph nodes, intensity-modulated radiotherapy (imrt)

## Abstract

Introduction: The use of intensity-modulated radiation therapy (IMRT) in head and neck cancers has allowed for selective sparing of low-risk or uninvolved lymph nodes. In oropharyngeal cancers, the benefits and risks of omitting contralateral retropharyngeal lymph nodes (RPLN) remain uncertain. This study examines the outcomes of elective coverage of contralateral RPLN in oropharyngeal cancer treated with definitive IMRT.

Methods: We analyzed 54 patients with newly diagnosed unilateral tonsil or base of tongue squamous cell carcinoma with at most unilateral neck involvement (cN0-N2b) and no RPLN involvement. These patients had no prior head and neck irradiation and were treated with definitive radiotherapy or chemoradiotherapy between 2012 and 2017. Cumulative incidences of local/regional/distant failure were estimated using competing risks methodology, and overall survival (OS) was estimated using the Kaplan-Meier method.

Results: All patients received elective nodal coverage to the ipsilateral RPLN, and 38 (62%) patients did not receive elective treatment of the contralateral RPLN. There were no significant differences in baseline characteristics. There were no contralateral RPLN failures observed. When comparing patients who received contralateral RP treatment with those who did not, there were no significant differences in two-year local failure (23% vs. 9%, p = 0.09), regional failure (18% vs. 4%, p = 0.12), or distant failure (15% vs. 9%, p = 0.62). Two-year OS was 89%. Mean parotid dose was not significantly lower after sparing vs. treating the contralateral RPLN (median 25.6 vs. 32.7 Gy, p = 0.15).

Conclusions: The omission of contralateral RPLN irradiation in tonsil or tongue base carcinomas with unilateral neck involvement is safe without compromising disease control.

## Introduction

The use of intensity-modulated radiation therapy (IMRT) in the treatment of head and neck squamous cell carcinoma (HNSCC) has become the standard of care over the past 15 years [1]. Compared to more traditional treatment approaches (conventional and conformal radiotherapy), IMRT may allow for better dose distribution that minimizes both acute and long-term toxicities, such as xerostomia and dysphagia, while still maintaining locoregional control of the target [2-3]. In IMRT the target volume and nontumor tissues are first identified on planning CT, and then doses to the tumor, as well as maximum dose acceptable to surrounding normal structures, such as the parotid glands and spinal cord, are determined. The selective nature of IMRT allows radiation oncologists to irradiate lymph node levels more likely to be involved with subclinical disease while sparing adjacent structures.

The retropharyngeal lymph nodes (RPLN) were first classified by Henri Rouviere in 1932 and are classified as part of level VIIa of the prevertebral compartment group [4]. This region is considered to have high rates of nodal metastasis from primary head and neck cancer, such as oropharyngeal (up to 20%-50% of soft palate or posterior pharyngeal wall), nasopharyngeal (up to 40%-90%), and hypopharyngeal cancers (up to 18%-57%), to warrant elective or therapeutic treatment [5]. While inclusion of the RPLN as IMRT targets in oropharyngeal cancer cases has been recommended [6], other studies [7-8] have shown that for patients with unilateral HNSCC, sparing the contralateral RPLN (the clinically uninvolved side of the neck) is associated with minimal risk of failure in these regions and resulted in greater quality of life.

The purpose of this study is to examine the outcomes of elective coverage of the contralateral RPLNs in patients with oropharyngeal carcinoma with at most unilateral neck involvement of treated with definitive IMRT. We also aim to assess the impact of this treatment decision on contralateral parotid radiation exposure.

## Materials and methods

This work was approved by the Institutional Review Board of Wake Forest University (IRB00047017), which permitted waiver of consent due to the retrospective nature of this study and the minimal risk to patients. In this study, 63 patients with newly diagnosed oropharyngeal SCC with at most unilateral neck involvement (cN0-N2b) without upfront retropharyngeal (RP) involvement treated with definitive radiotherapy or chemoradiotherapy between 2012 and 2017 were identified. Exclusion of patients with soft palate and posterior pharyngeal wall primary sites (*n* = 3) or those without evaluable radiotherapy treatment plans (*n* = 6) yielded 54 patients to be included in the final analysis. Patients with primary tumors crossing midline were not specifically excluded. Patient and treatment characteristics such as age, gender, Eastern Cooperative Oncology Group (ECOG) performance status, primary tumor site, clinical stage, tumor human papillomavirus (HPV) status, radiotherapy dose/fractionation, and utilization of chemotherapy were abstracted from the medical record.

Patient workup included clinical evaluations by otolaryngology, radiation oncology, medical oncology, speech language pathology, and dietetics in addition to discussion with pathologists and radiologists as part of a multidisciplinary head and neck cancer group. Primary disease staging included flexible fiberoptic nasopharyngolaryngoscopy, contrast-enhanced computed tomography (CT) imaging of the head/neck and chest, or positron emission tomography (PET/CT). Staging was completed according to the American Joint Commission on Cancer (AJCC) seventh edition [9]. All patients were treated with conventionally fractionated radiotherapy as described below. Concurrent chemotherapy was delivered at the discretion of the treating medical oncologist using a variety of mostly cisplatin- or carboplatin-based regimens. Radiotherapy planning was performed using CT simulation with or without contrast with thermoplastic mask immobilization. Gross tumor was delineated and expanded by 0.5-1 cm to form a clinical target volume (CTV) defined as CTV70 to receive 70 Gray (Gy), the high-risk involved and/or elective ipsilateral neck were defined within a CTV to receive 63 Gy (CTV63), and the low-risk/uninvolved neck was defined as a CTV to receive 56 Gy (CTV56). Planning target volumes (PTV) were generated by expanding each respective CTV by 0.3–0.5 mm to form planning target volumes to receive 70 (PTV70), 63 (PTV63), and 56 Gy (PTV56). Radiotherapy was delivered daily, at doses of 2 Gy per fraction to PTV70, 1.8 Gy per fraction to PTV63, and 1.6 Gy per fraction to PTV56 using a simultaneous integrated boost technique.

Radiotherapy treatment plans were retrospectively reviewed and evaluated for coverage of the RPLN basins as previously defined [4]. Treatment of this region was at the discretion of the treating radiation oncologist and dependent upon various risk factors including primary tumor site, laterality, and involved lymph node burden/location. Treatment of the contralateral RP nodal basin was defined as inclusion of this region within one of the nodal CTVs as described above. Mean dose to the ipsilateral and contralateral parotid gland were also abstracted from the treatment planning software.

Patients were seen within four to six weeks post-treatment and followed clinically every two to three months thereafter. CT or PET/CT imaging was obtained two to three months post-treatment and patients were followed with clinical examination and radiography at the discretion of the treating clinician thereafter. Disease recurrence was defined as any radiographic or pathologic evidence of disease in the primary site (local), neck (regional), or areas outside the neck (distant). Potential dysphagia-related toxicities at six months post-treatment were collected, including percent weight change from baseline and gastrostomy tube-dependence, defined as any proportion of a patient's daily enteral nutrition via tube.

Descriptive analyses were performed using count (frequency) and mean/median (range/interquartile range, IQR) for categorical and continuous normal and non-normal variables, respectively. Categorical variables were compared across strata using the Fisher's exact and Chi-square tests as appropriate; continuous variables were compared using t-test and Wilcoxon rank sum test. Time-to-event estimates were generated from the time of diagnosis. Survival was estimated using the Kaplan-Meier method. Cumulative incidence (CI) of local, regional, and distant failure was estimated using death without failure as a competing risk. Statistical analyses were performed using R version 3.5 (R Foundation for Statistical Computing, Vienna, Austria).

## Results

Of the 63 identified patients, 54 met criteria for inclusion in the final analysis. Patient baseline and treatment characteristics are depicted in Table 1. All patients received elective nodal coverage to the ipsilateral RP basin; 17 received contralateral RP coverage and 37 did not. There were no differences in baseline characteristics between patients with contralateral RP nodes treated vs. untreated. Upon review of the individual treatment plans, 53 of 54 patients had gross tumor volume within 1 cm of midline. HPV-associated disease was present in 80%. Median follow-up was 24.3 months (95% CI 18.6-42.1). Two patients with stage II disease and one with stage III disease received radiotherapy alone.

**Table 1:** Patient and treatment characteristics. ECOG: Eastern Cooperative Oncology Group; HPV: human papillomavirus; *n*: number.

	Total (*n* = 54)	No contralateral RPLN treatment (*n* = 37)	Contralateral RPLN treatment (*n* = 17)	p-value
Age, mean (range)	58 (41-78)	58 (42-72)	61 (41-78)	0.34
Gender, *n* (%)				
Male	47 (87)	31 (84)	16 (94)	0.41
Female	7 (13)	6 (16)	1 (6)	
ECOG performance status, *n* (%)				
0	19 (35)	11 (30)	8 (47)	0.24
1	31 (57)	24 (65)	7 (41)	
2	4 (7)	2 (5)	2 (12)	
Primary site, *n* (%)				
Tonsil	26 (48)	20 (54)	6 (35)	0.20
Base of tongue	28 (52)	17 (46)	11 (65)	
T stage, *n* (%)				
T1	9 (17)	6 (16)	3 (18)	0.92
T2	28 (52)	20 (54)	8 (47)	
T3	10 (19)	7 (19)	3 (18)	
T4a	7 (13)	4 (11)	3 (18)	
N stage, *n* (%)				
N0	7 (13)	6 (16)	1 (6)	0.24
N1	6 (11)	2 (5)	4 (24)	
N2a	4 (7)	3 (8)	1 (6)	
N2b	37 (69)	26 (70)	11 (65)	
Clinical stage, *n* (%)				
II	3 (6)	3 (8)	0 (0)	0.16
III	7 (13)	3 (8)	4 (24)	
IVA	44 (81)	31 (84)	13 (77)	
HPV-positive, *n* (%)	41 (80)	27 (77)	14 (88)	0.47
Concurrent chemotherapy, *n* (%)	51 (94)	35 (95)	16 (94)	1.00
Type of chemotherapy, *n* (%)				
Cisplatin	42 (76)	30 (86)	10 (63)	0.10
Carboplatin/paclitaxel	7 (13)	2 (6)	4 (25)	
Platinum/cetuximab	1 (2)	1 (3)	0 (0)	
Docetaxel	1 (2)	0 (0)	1 (6)	
Other	4 (7)	2 (6)	1 (6)	
Induction chemotherapy, *n* (%)	3 (5)	2 (5)	1 (6)	1.00

**Figure 1:**
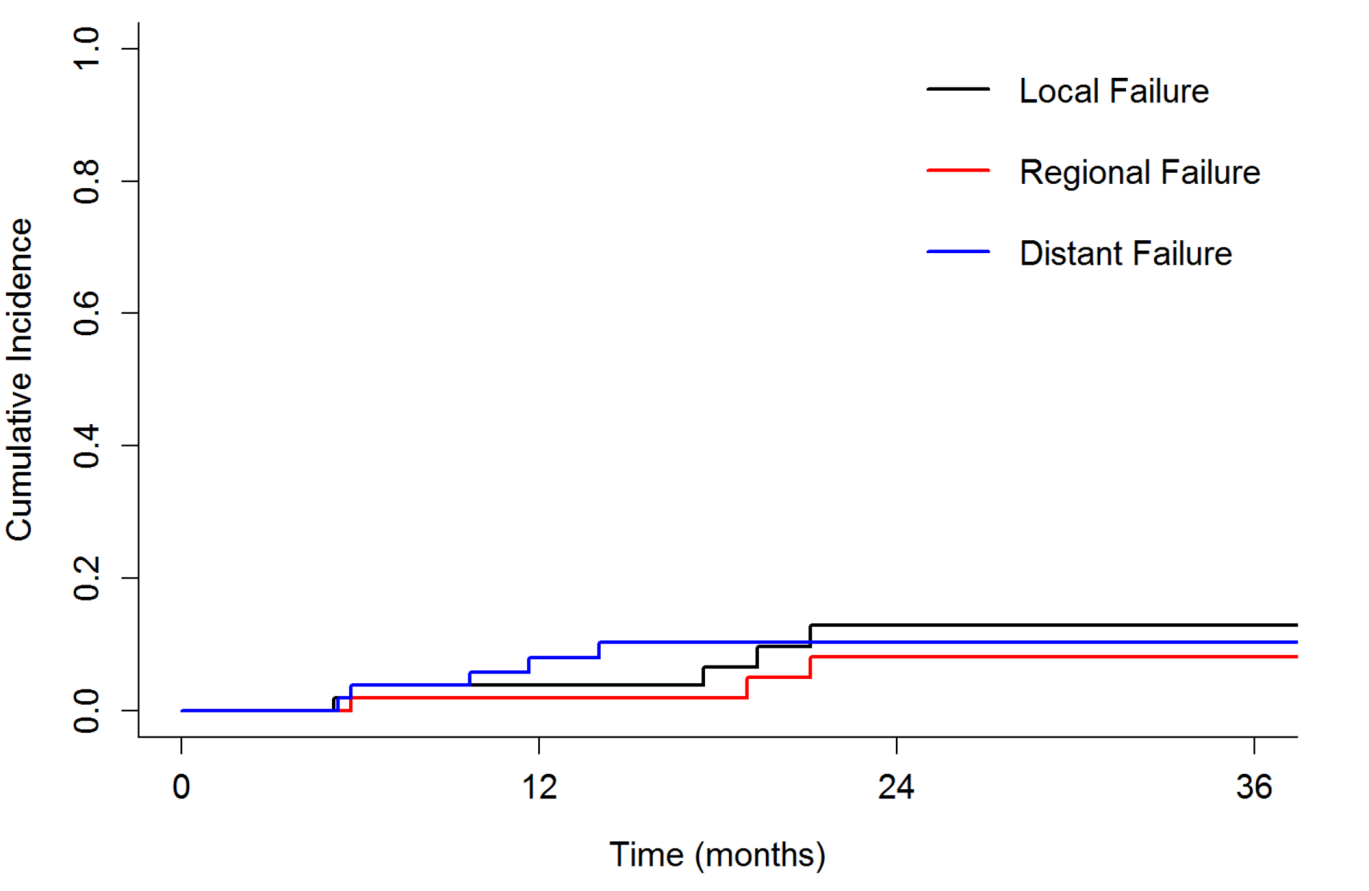
Cumulative incidence of local failure (black), regional failure (red), and distant failure (blue).

**Figure 2:**
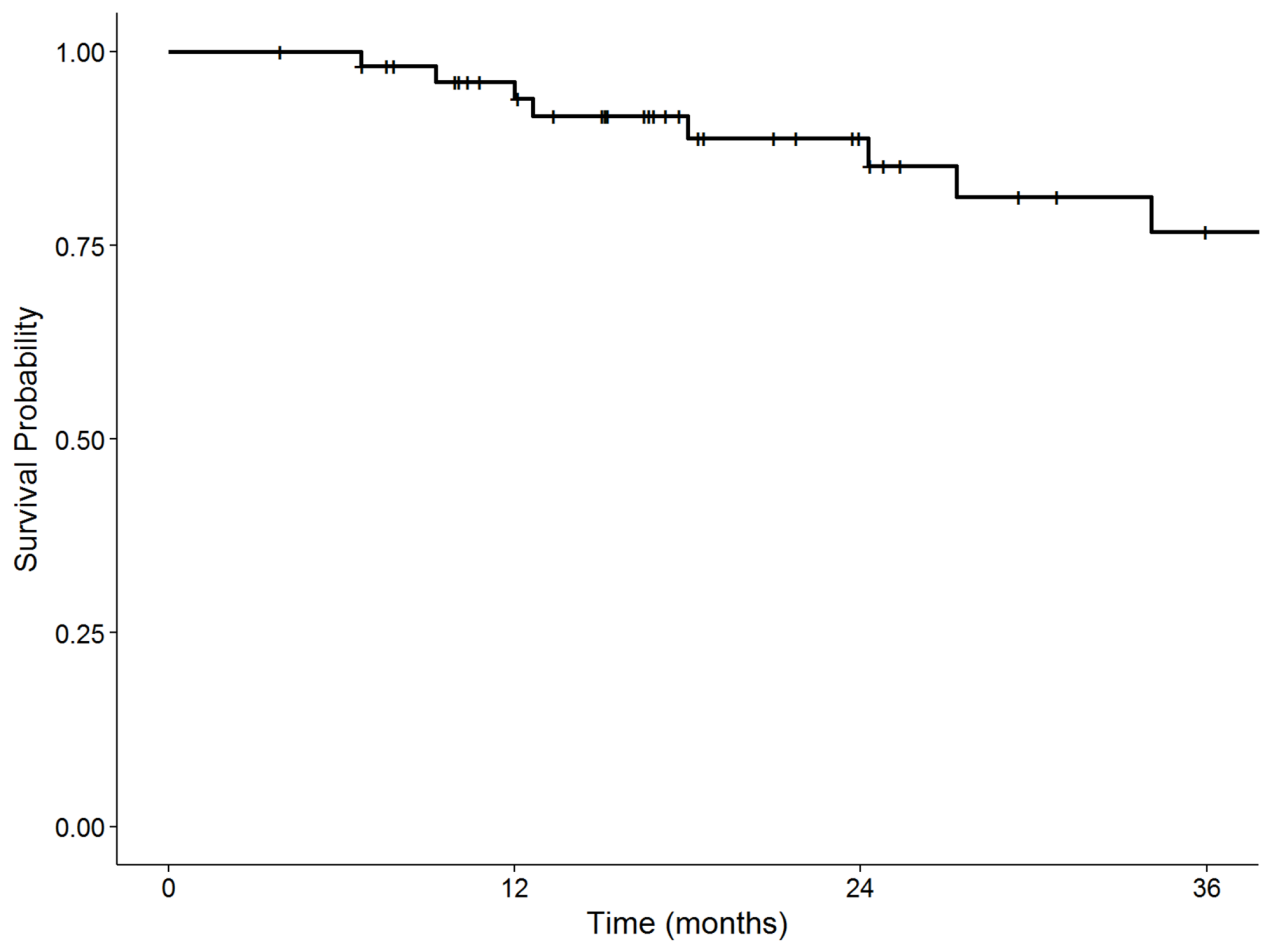
Kaplan-Meier plot of overall survival.

No RPLN failures occurred. Cumulative incidence of local, regional, and distant failure at two years was 13%, 8%, and 10%, respectively (Figure 1). No contralateral neck failures were observed. Comparing patients who received contralateral RPLN irradiation vs. those who did not, there were no significant differences in two-year CI of local failure (23% vs. 9%, p = 0.09), regional failure (18% vs. 4%, p = 0.12), or distant failure (15% vs. 9%, p = 0.62). The two-year CI of locoregional failure was 10% in the HPV-positive patients vs. 25% in HPV-negative patients (p = 0.14). Two-year overall survival was 89% (Figure 2). Two-year disease-free survival was 78%.

In total, three patients with initial locoregionally advanced disease experienced regional failure, and all were located within the irradiated ipsilateral level II. A 64-year-old male with T3N2b HPV-negative base of tongue carcinoma was treated with 70 Gy in 35 fractions with concurrent cetuximab and experienced simultaneous local and ipsilateral level II in-field failure (within PTV70) with synchronous lung metastases at six months. A 41-year-old female with T2N2b HPV-positive base of tongue carcinoma who received 70 Gy with concurrent cisplatin (100 mg/m^2^ every three weeks) developed synchronous local and regional (within PTV70) failure at approximately 18 months. A 49-year-old male with T3N2a HPV-positive SCC of the tonsil treated with 70 Gy with concurrent cetuximab developed lung metastases at nine months post-treatment followed by synchronous local and regional (within the PTV63) progression at 21 months.

The median value of the mean dose to the contralateral parotid was 25.62 Gy (interquartile range, 24.40-33.48) in the patients without contralateral RP treatment compared to 32.69 Gy (IQR 25.71-36.45, p = 0.15). There was no difference in the mean dose to the ipsilateral parotid between groups (median 42.03 Gy, IQR 36.57-52.34). Mean weight change at six months was –13% (range –40% to +2%) and 19% of patients were gastrostomy tube-dependent at six months; there were no differences between groups for either of these outcomes. 

## Discussion

Retropharyngeal lymph node metastases, identified in 13%-41% of patients in select surgical series, represent a significant risk to patients with oropharyngeal carcinoma [10-11]. Radiographic detection of pathologic RP lymph node metastases occur in approximately 10%-21% of patients and portend a poorer survival in comparison with patients with no radiographic evidence of RPLN involvement [12-13]. Since the adoption of conformal radiotherapeutic techniques such as IMRT for the treatment of head and neck cancers, guidelines developed to aid the treating radiation oncologist in defining the appropriate lymph node basins at risk advocate for the coverage of the bilateral RPLN regions for oropharyngeal cancers [14].

A prospective study [7] of patients with a variety of head and neck primary carcinomas treated with IMRT at Washington University between 1997 and 2010, during which three paradigms of neck nodal coverage were utilized. This study included patients with AJCC seventh edition stage I-IVB disease, most frequently involving the oropharynx, 64% of whom underwent upfront surgical resection. Initially, bilateral neck and RPLN were treated electively with successive omission of the contralateral level II and then the contralateral RPLN. While there were two patients with failures in the ipsilateral RPLN, no contralateral RP or level II failures were observed. Additionally, quality of life metrics measured using the MD Anderson Dysphagia Index were significantly improved in the group with omission of the contralateral RPLN and level II lymph node basins as compared to those patients who received comprehensive bilateral neck irradiation. Our findings are consistent with this report, as all three regional failures occurred within the ipsilateral neck, an area surely covered with this paradigm. Additionally, two of the three patients were treated with concurrent cetuximab, a regimen now known to be associated with poorer disease control in comparison to cisplatin chemoradiotherapy [15-17].

Another recent study [8] reviewed 102 patients with oropharyngeal carcinoma with disease limited to the ipsilateral neck (cN0-2b) treated with IMRT. With a majority (94%) HPV-associated disease, control outcomes were excellent, and no contralateral RPLN failures occurred. In the current series, 80% of patients were HPV-positive, and 2-year locoregional control in the HPV-positive cohort was comparable.

While the mean dose to the contralateral parotid was slightly less in the group who did not receive contralateral RPLN treatment, this did not reach statistical significance. Additionally, there were no significant changes in weight loss or gastrostomy tube dependence at six months. This is likely related to limited sample size, consistent coverage of the contralateral level II in all patients, and the multifactorial indications for gastrostomy tube support. Nevertheless, the results of these studies suggest that patients may be spared the potential morbidity of contralateral RPLN treatment without compromising disease control. Additionally, sparing the contralateral RPLN may lessen the dose to other adjacent normal structures such as the pharyngeal constrictors, potentially reducing the risk of gastrostomy tube-dependence and late radiation-associated dysphagia as shown in other studies [18-20]. Since the pharyngeal constrictors were not routinely defined in the treatment planning process for this cohort, information regarding radiation dose to these structures is not available. Conclusions regarding dose limitations to prevent radiation-associated dysphagia are difficult to make with the current level of evidence; further reporting of consistent swallowing measures/outcomes is required [21].

These findings are limited by the selection bias inherent in retrospective patient chart reviews, as well as potential variations in clinical practice such as frequency of surveillance imaging over the course of follow-up. Additionally, this study included a limited sample size of patients with oropharyngeal squamous cell carcinoma with ≤cN2b disease, as patients with contralateral disease are generally considered at higher risk of RPLN recurrence. This limits these findings to this relatively select group of patients, but patient selection is paramount when omitting treatment to potential regions at risk for spread of disease. Since this particular clinical scenario is relatively uncommon, the sample size of each cohort size is small, limiting our power to assess for any difference between the groups. This limits the conclusions made herein to hypothesis generation. Larger, pooled analyses of the risk of contralateral RPLN recurrence after omission of elective irradiation may serve to better answer this key clinical question.

## Conclusions

In conclusion, based on our findings, omission of the contralateral retropharyngeal lymph node region in patients with tonsillar and base of tongue squamous cell carcinomas with up to unilateral neck involvement is safe and yields comparable locoregional control.
